# What are the implications of sex differences in cell death for treatment after neonatal hypoxia-ischemia, if any?

**DOI:** 10.1038/s41390-025-04134-6

**Published:** 2025-05-20

**Authors:** Alice McDouall, Thomas R. Wood, Benjamin A. Lear, Joanne O. Davidson, Laura Bennet, Alistair J. Gunn, Justin M. Dean

**Affiliations:** 1 Department of Physiology, The University of Auckland, Auckland, New Zealand; 2 Department of Pediatrics, University of Washington, Seattle, WA USA

Our understanding of the mechanisms by which cells die during and after neonatal hypoxic-ischemic (HI) brain injury continues to be elucidated, and currently involves a wide range of overlapping pathways that include necrosis, apoptosis, necroptosis, ferroptosis, and autophagy.^1^ The predominant pathway seems to depend on several factors, including the severity of HI, the stage in the evolution of injury, the developmental age, and the availability of adenosine triphosphate.^2^ Importantly, there is evidence of a sexual dimorphism in the predominant cell death pathway after HI. For example, in the Rice−Vannucci murine model of HI brain injury, there is extensive evidence that cell death primarily occurs via caspase-3 dependent pathways in females,^3,4^ whereas in males it occurs via caspase-independent, poly [adenosine diphosphate ribose] polymerase 1 (PARP-1) dependent pathways.^4,5^ In this volume, Alonso-Alconada et al. confirm that this sex difference is not unique to rodents and is also seen in a piglet model of neonatal brain injury. In that study, although necrosis was the predominant form of cell death in both sexes, female piglets exposed to carotid artery occlusion and hypoxia had more apoptotic cell death than males, who had an increased prevalence of necrotic cell death, at 48 hours after HI.^6^ The underlying mechanisms of these differences between males and females are unclear, but likely include differences in the vulnerability to oxidative stress, production of reactive oxygen species, and mitochondrial dysfunction.^7^ Furthermore, speculatively given that overall cell loss was greater in males than females in the piglet study, potentially this drove more cell death through the necrotic pathway.

Despite these apparent differences in cell death pathways between males and females, it is important to consider whether this results in any meaningful difference in the treatment of HI brain injury. In principle, treatments specifically aimed at reducing a certain pathway of cell death could be exclusively given to males or females, if one is more likely to benefit from the intervention. For example, treatment with Necrostatin, an inhibitor of programmed cell necrosis through receptor-interacting serine threonine protein kinase 1 (RIP-1) activation, was associated with reduced infarct size specifically in male rats, but not females, exposed to carotid artery occlusion and hypoxia.^8^

Sex differences in cell death pathways may in part contribute to the sex-dependent effects of therapeutic hypothermia observed in some pre-clinical studies. For example, in a large retrospective meta-analysis of studies from a single laboratory, hypothermia following HI was only neuroprotective in female and not male rats.^9^ In another study, female rats treated with hypothermia had a marked reduction in brain damage and a significant improvement in functional score, which was not seen with both sexes combined.^10^ One possible explanation is that female rats are more likely to be cooler than male rats when they are returned to their nests following hypothermia treatment, potentially meaning they have an extended cooling duration compared with males.^11^ In line with this, in a large rat meta-analysis, greater body weight was associated with more severe injury in the hypothermia group but not the normothermia group. Finally, it is important to appreciate that both these studies used durations of hypothermia that are much shorter than what is employed clinically or in large animal models, which may confound the interpretation of these results.

Strikingly, the NICHD and CoolCap trials found no significant effect of sex on response to therapeutic hypothermia in infants with moderate-severe hypoxic-ischemic encephalopathy.^12,13^ These trials were arguably underpowered, but the preliminary analysis of a recent large meta-analysis of the original and more recent clinical trials involving therapeutic hypothermia has reported no effect of sex.^14^ These findings are likely because hypothermia has very broad actions, and therefore suppresses both apoptotic and necrotic programmed cell death pathways.^15^ This is important as, even though Alonso-Alconada et al. show greater apoptotic cell death in female piglets, necrosis was the primary mode of cell death in both sexes. Therefore, at least for treatment with hypothermia in humans, even if there is sexual dimorphism in the balance of pathways of cell death, the majority of cells are likely to die through similar mechanisms, and so there seems to be convergence such that hypothermia improves outcomes similarly in both males and females.

Where sexual dimorphism might have a greater impact clinically is with add-on or alternative treatments for hypothermia. For example, in some studies, the neurotrophic factor erythropoietin was shown to only improve outcomes in female, but not male, rats after exposure to neonatal carotid artery ligation and hypoxia.^16,17^ Again, by contrast, the High-Dose Erythropoietin for Asphyxia and Encephalopathy (HEAL) trial of erythropoietin treatment in conjunction with hypothermia in term infants with hypoxic-ischemic encephalopathy reported no sex difference in outcomes.^18^ It is unclear whether this reflects limitations of translating from rodents to humans, or in part is due to hypothermia’s very broad range of actions.^19^ It should also be noted that other alternative treatments to hypothermia in development, such as umbilical cord blood cells and melatonin, do not currently show any sexual dimorphism in outcomes.^20,21^

To conclude, the study by Alonso-Alconada et al. adds to the substantial preclinical evidence for sexual dimorphism in the pathways leading to cell death after HI.^6^ This reinforces the importance of including both male and female animals in preclinical studies, as they were in the foundational fetal sheep studies of therapeutic hypothermia,^22–24^ even if the studies are not powered to allow for separation by sex. Thus, the historic notion that females can be excluded from studies to minimize variability is both untrue and unacceptable.^25,26^ This flawed approach may result in treatments being prematurely dismissed, as well as limiting the translatability of potential neuroprotective treatments. Nevertheless, the clinical evidence that therapeutic hypothermia is equally effective in males and females is reassuring.
